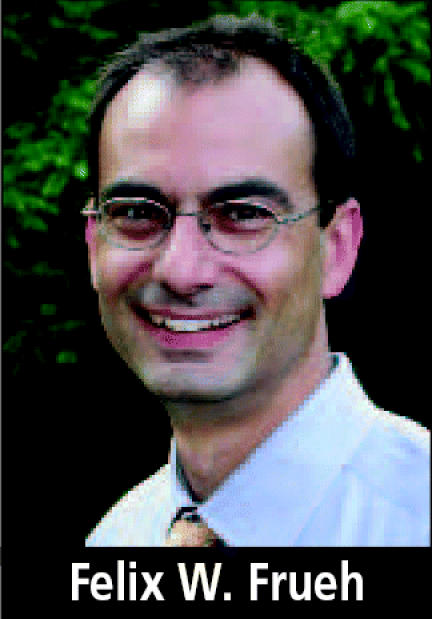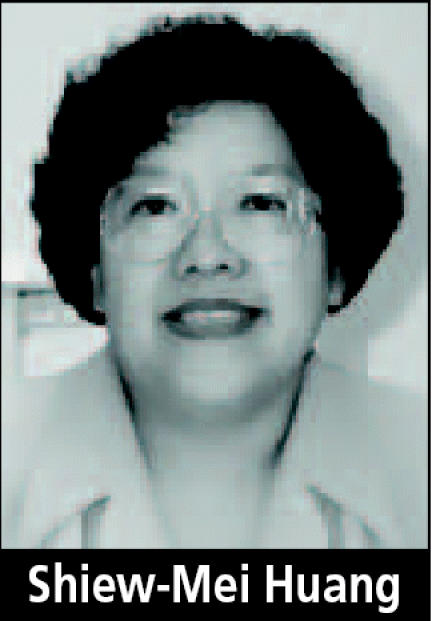# Guest Editorial: Regulatory Acceptance of Toxicogenomics Data

**DOI:** 10.1289/ehp.112-1277121

**Published:** 2004-08

**Authors:** Felix W. Frueh, Shiew-Mei Huang, Lawrence J. Lesko

**Affiliations:** Office of Clinical Pharmacology and Biopharmaceutics, Center for Drug Evaluation and Research, U.S. Food and Drug Administration, Rockville, Maryland E-mail: huangs@cder.fda.gov

Early identification of toxicologic side effects of a drug candidate is critical to an efficient drug discovery and development process. Toxicogenomics, the marriage of data-rich genomics approaches with traditional toxicologic end point evaluation combined with increasingly powerful *in silico* modeling approaches, promises to accelerate this process. The advent of parallel experimental platforms, for example, DNA microarrays, has enabled us to gain insight into complex biologic responses to drugs. The challenge is to analyze and correctly interpret these large data sets. Currently, no common standards exist for such data even though attempts are being made to streamline and standardize the presentation of the information. These efforts include ArrayExpress infrastructure for microarray data (http://www.ebi.ac.uk/arrayexpress), Minimum Information About a Microarray Experiment (http://www.mged.org/Workgroups/MIAME/miame.html), and MicroArray Gene Expression (MAGE) markup language (http://www.mged.org; http://www.omg.org/technology/documents/formal/gene_expression.htm).

The creation of vast amounts of genomics and toxicogenomics data has sparked the development of novel systems to handle this type of information. Ultimately, the success of a toxicogenomics approach in drug development depends on our ability to interpret the data in relation to existing information (e.g., screening of a drug-induced gene expression fingerprint against a database containing drug-related gene expression toxicity profiles). It is critical that interdisciplinary information (chemistry, biochemistry, genetic, genomics, clinical) be integrated into the same data warehouse. Incorporating toxicogenomics data into this approach, which is often referred to as systems biology, will help us understand in much more depth how cells maintain homeostasis and how organisms respond to drug exposure at the molecular level.

“. . . the identification, verification, and validation of biomarkers are critical components of every pharmacogenomics, as well as toxicogenomics, study of cases in regulatory decision making.”

The mission of the U.S. Food and Drug Adminstration (FDA) states that the agency “. . . is responsible for advancing the public health by helping to speed innovations that make medicines and foods more effective, safer, and more affordable. . . .” (FDA 2004). Former agency commissioner Mark McClellan stated that “the FDA priority is facilitating the use of pharmacogenetics-driven treatments” (Salerno and Lesko 2004). The FDA has recently issued a draft, “Guidance for Industry: Pharmacogenomic Data Submissions” (FDA 2003), and has held workshops to discuss issues related to pharmacogenomics data submissions (Salerno and Lesko 2004a, 2004b; Leighton et al. 2004; Ruaño et al. 2004; Trepicchio et al. 2004). This guidance is being revised on the basis of public comments, and a final guidance should be issued later this year. Many principles found in this guidance apply to toxicogenomics studies. In particular, the identification, evaluation, and validation of biomarkers are critical components of every pharmacogenomics, as well as toxicogenomics, study of cases in regulatory decision making. The guidance is general and includes examples of genetic and genomic biomarkers: a *CYP2D6* (cytochrome P450 2D6) mutation versus an increase in HER2 (human epidermal growth factor receptor 2) expression can be viewed as genetic and genomic biomarkers, respectively. However, it is anticipated that future data submissions will contain many more complex gene expression profiles and large-scale single nucleotide polymorphism maps (e.g., from whole genome scans), which will present new challenges to define the analytical and clinical validity of such new and highly complex bio-marker sets. The guidance represents the FDA’s current view on pharmacogenomics and what the agency believes are the scientific grounds for evaluating such information as it relates to voluntary versus required submission of data.

What are the next steps? Regulators have been criticized for the lack of guidance in the new era of genomics-based drug development. In addition to the guidance on pharmacogenomics data submissions (FDA 2003), the FDA is embarking on a new guidance initiative for the co-development of pharmacogenomics-based drugs and biologic products and the diagnostic tests necessary for therapeutic decision making. Recently, the FDA and the Drug Information Association (DIA) sponsored a pharmacogenomics workshop (FDA/DIA 2004). The purpose of the workshop was to identify issues in the development of pharmacogenomics-based combination products. We hope to see the base of pharmacogenomics knowledge grow and expand, and we look forward to the use of this information in the drug discovery and regulatory evaluation processes. We expect that not only the novel scientific but also the newly created regulatory tools such as voluntary submissions of genomics data will provide the means by which genomics-based research can excel in advancing public health and drug development.

## Figures and Tables

**Figure:**